# Forced expression of S100A10 reduces sensitivity to oxaliplatin in colorectal cancer cells

**DOI:** 10.1186/1477-5956-12-26

**Published:** 2014-05-09

**Authors:** Sayo Suzuki, Yusuke Tanigawara

**Affiliations:** 1 Department of Clinical Pharmacokinetics and Pharmacodynamics, School of Medicine, Keio University, 35 Shinanomachi, Shinjuku-ku, Tokyo 160-8582, Japan; 2 Center for Pharmacy Practice, Faculty of Pharmacy, Keio University, 1-5-30 Shibakoen, Minato-ku, Tokyo 105-8512, Japan

**Keywords:** S100A10, Oxaliplatin, Annexin A2, Colorectal cancer

## Abstract

**Background:**

Individual responses to oxaliplatin (L-OHP)-based chemotherapy remain unpredictable. Our recent proteomics studies have demonstrated that intracellular protein expression levels of S100A10 are significantly correlated with the sensitivity of colorectal cancer (CRC) cells to L-OHP, but not 5-FU, suggesting that S100A10 is a candidate predictive marker for the response to L-OHP. In this study, we investigated whether S100A10 is involved in L-OHP sensitivity or not.

**Results:**

Forced expression of S100A10 in COLO-320 CRC cells significantly increased the 50% inhibitory concentration (IC_50_) for L-OHP (*P* = 0.003), but did not change that for 5-FU, indicating that S100A10 is more specific to L-OHP than 5-FU. Silencing of the S100A10 gene showed no apparent effect on sensitivity to L-OHP in HT29 cells. Silencing of the annexin A2 (a binding partner of S100A10) gene alone downregulated both annexin A2 and S100A10 protein levels, with no change in S100A10 gene expression. However, original levels of intact S100A10 protein in CRC cells positively correlated with S100A10 mRNA levels (*P* = 0.002, *R* = 0.91).

**Conclusions:**

The present results have shown that protein expression of S100A10 was associated with resistance to L-OHP, but not 5-FU, supporting the hypothesis that S100A10 expression may predict L-OHP sensitivity. Thus, our present study provides basic findings to support that S100A10 expression can be used as a predictive marker for tumor sensitivity to L-OHP.

## Background

Oxaliplatin (L-OHP) is a key drug used for the treatment of colorectal cancer (CRC) [1]. L-OHP and bolus/infusional 5-fluorouracil (5-FU) combined with folinic acid (FOLFOX) have yielded high response rates (~50%) and good overall survival [2-4]. Recently, this regimen has emerged as one of the most effective therapeutic regimens available, providing a platform for the treatment of CRC [5-7]. However, approximately half of all patients who receive FOLFOX gain no benefit, despite the usual risk of toxicity. Predictive markers of the response to L-OHP have not yet been established. Although several predictive markers of the response to platinum-based chemotherapy have been proposed on the basis of various mechanisms of chemoresistance to platinum drugs [8], the UK MRC FOCUS (Fluorouracil, Oxaliplatin, CPT-11: Use and Sequencing) clinical trial, the largest randomized biomarker trial in metastatic CRC to date, reported no significant association between response to platinum-based chemotherapy and excision repair cross-complementing rodent repair deficiency, complementation group 1 (*ERCC1*)*,* xeroderma pigmentosum group D (*XPD*, also known as *ERCC2*)*,* glutathione-*S*-transferase-P1 (*GSTP1*), or other candidate biomarkers that have previously shown promise [9].

Recently, our proteomics studies have demonstrated that intracellular S100A10 protein expression levels are significantly correlated with the sensitivity of CRC cells to L-OHP, but not 5-FU, providing a new candidate predictive markers for the response to L-OHP [10]. S100A10 is a member of the S100 family of proteins. It has been shown to interact with a variety of proteins, including plasma membrane-resident receptors [11-14], indicating that S100A10 is an active regulator and/or is involved in the trafficking of cellular/membrane proteins which lead to various biological functions. S100A10 mRNA, S100A10 protein, or both have been found in many types of cells, tissues, and tumors [15-21]. S100A10 has also attracted considerable attention for its role as an essential molecule in tumor progression via macrophage migration to tumor sites [22]. In addition, most of the S100A10 protein is tightly associated with dimers of annexin A2, forming an (S100A10)_2_-(annexin A2)_2_ heterotetramer [23-25]. Annexin A2 is a member of the annexin family which has been reported to have multiple functions [26-29]. However, the mechanisms of S100A10 involvement in chemoresistance, including the possible participation of annexin A2, are still unknown.

In this study, we investigated whether alterations in S100A10 and/or annexin A2 expression were involved in mediating chemosensitivity to L-OHP by using forced overexpression of S100A10 in stably transfected cells and RNA interference. Our results have demonstrated the potential contribution of S100A10 to resistance to L-OHP.

## Results

### Effects of forced expression of S100A10 on cell proliferation and sensitivity to L-OHP or 5-FU in COLO-320 cells

**Figure 1:**
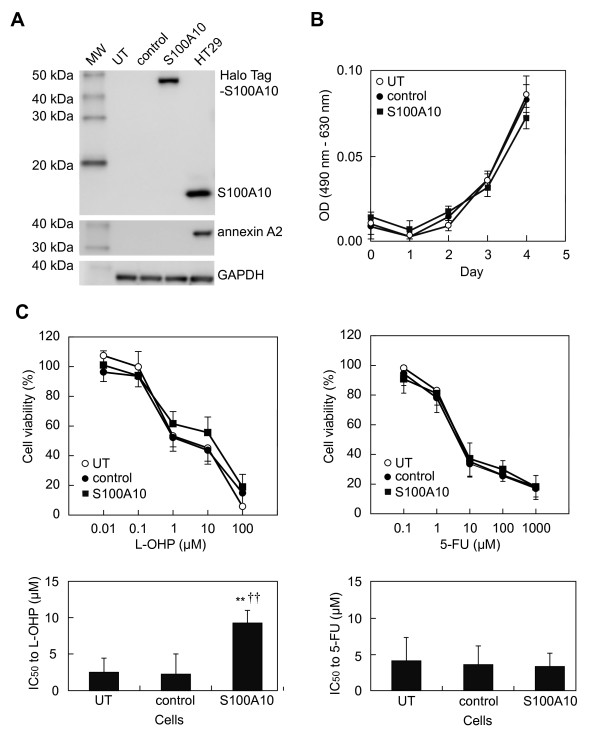
**Effects of forced expression of S100A10 on cell proliferation and chemosensitivity. (A)** Untransfected COLO-320 cells (UT), cells transfected with non-target vector (control), cells stably transfected with S100A10 (S100A10), and HT29 cells used as a positive control were lysed. Cell lysates (10 μg protein) were subjected to western blot analysis using anti-S100A10 (1:5000), anti-annexin A2 (1:2500), and anti-GAPDH antibodies (1:4000). Molecular weight standards (MW) are in the left lane. **(B)** The growth curves of each cell line. **(C)** Each cell was treated with various concentration of L-OHP or 5-FU. Cell viability after exposure to L-OHP or 5-FU (upper panel), and IC_50_ values for L-OHP or 5-FU (lower panel) were determined. Data are the mean ± SD (n = 4). *P* values are by comparison with the results for UT (**P < 0.01) and control (††, *P* < 0.01) by on-way ANOVA with Tukey’s post hoc test.

Stably transfected COLO-320 cells expressing S100A10 (COLO-320/S100A10) showed strong expression of S100A10 (HaloTag-tagged S100A10) as a 44-kDa protein (S100A10: 11 kDa, HaloTag: 33 kDa). The level of expression was similar to that of endogenous S100A10 levels in HT29 cells, a line which shows high S100A10 protein expression. Annexin A2, the binding partner of S100A10, was not expressed in COLO-320/S100A10 cells (Figure 1A). The proliferation rates of COLO-320/S100A10, COLO-320/vector, and untreated COLO-320 cells were similar (Figure 1B). Cell viability after L-OHP or 5-FU exposure (Figure 1C, upper panel) and IC_50_ values (Figure 1C, lower panel) were determined. Forced expression of S100A10 significantly increased the IC_50_ value of COLO-320/S100A10 for L-OHP (9.3 ± 1.8 μM [mean ± SD]) compared to that for the COLO-320/vector (2.3 ± 2.0 μM) and untreated COLO-320 cells (2.5 ± 1.8 μM; one-way ANOVA, *P* = 0.001; Tukey’s test, *P* = 0.003 and *P* = 0.002, respectively; Figure 1C, left panel), while that for 5-FU was unchanged. The IC_50_ values (μM; mean ± SD) of cells for 5-FU were as follows: COLO-320/S100A10, 3.4 ± 1.8; COLO-320/vector, 3.6 ± 2.6; and untreated COLO-320 cells, 4.1 ± 3.2 (Figure 1C, right panel).

### Effects of S100A10 or/and annexin A2 gene silencing by siRNAs on cell proliferation and sensitivity to L-OHP in HT-29 cells

**Figure 2:**
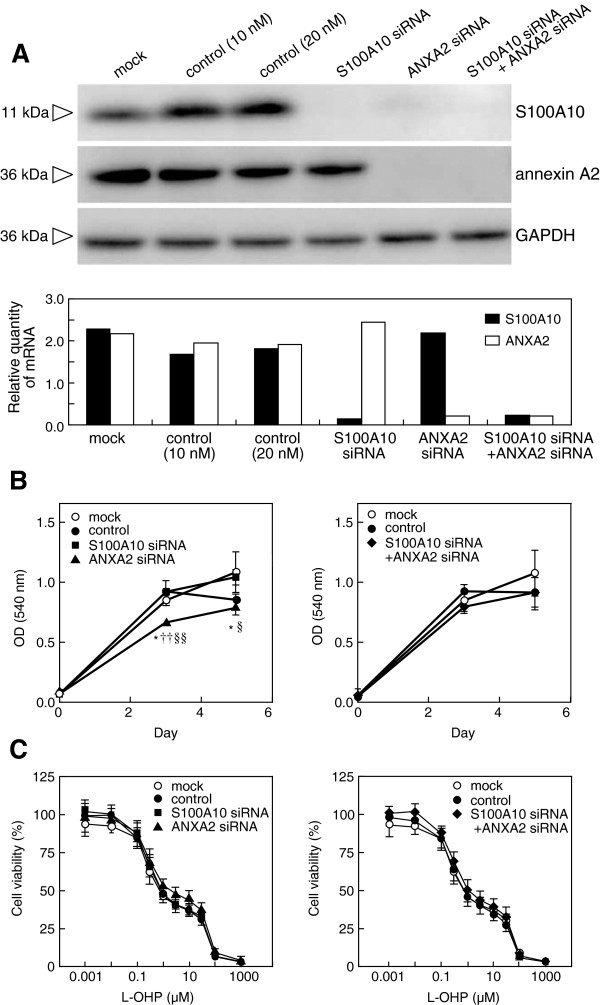
**Effects of S100A10 or/and annexin A2 gene silencing on cell proliferation and chemosensitivity.** HT29 cells were treated with transfection reagent alone (mock) or transfected with nontargeting control siRNA (control, final concentration 10 nM or 20 nM), S100A10 siRNA (S100A10 siRNA, final concentration 10 nM), annexin A2 siRNA (ANXA2 siRNA, final concentration 10 nM), or both S100A10 siRNA and annexin A2 siRNA (S100A10 siRNA + ANXA2 siRNA, final concentration 20 nM). **(A)** The expression levels of S100A10 and annexin A2 were examined by using real-time qRT-PCR (lower panel) and western blot analyses (upper panel). **(B)** Cell proliferation was determined after transfection for the indicated times (left panel, single knockdown; right panel, double knockdown). Data are the mean ± SD (n = 3). *P* values are by comparison with the results for the mock (*, *P* < 0.05), control (††, *P* < 0.01), or S100A10 siRNA (§, *P* < 0.05; §§, *P* < 0.01) by one-way ANOVA with Tukey’s post hoc test. **(C)** Cell viability after exposure to L-OHP was determined. Cells were treated with various concentrations of L-OHP after single knockdown with each siRNA (left panel) or after double knockdown of S100A10 and annexin A2 (right panel). Data are the mean ± SD (n = 9).

Real-time qRT-PCR analyses revealed efficient and specific suppression of S100A10 or/and annexin A2 mRNAs in HT29 cells (Figure 2A, lower panel). Western blot analyses also revealed the efficient suppression of protein expression of target molecules (Figure 2A, upper panel). However, silencing of the annexin A2 gene induced the downregulation of both annexin A2 and S100A10 protein levels, with no change in S100A10 gene expression (Figure 2A, upper panel).

Cells transfected with annexin A2 siRNA (HT29/siANXA2) exhibited slightly lower cell proliferation rates at day 3 compared to those for cells treated with transfection reagent alone (mock), cells transfected with nontargeting control siRNA (control), and cells transfected with S100A10 siRNA (HT-29/siS100A10) (ANOVA, *P* = 0.004; Tukey’s test, *P* = 0.031, *P* = 0.006, and *P* = 0.006, respectively; Figure 2B, left panel), and at day 5 compared to mock or HT-29/siS100A10 (ANOVA, *P* = 0.015; Tukey’s test, *P* = 0.023 and *P* = 0.049, respectively; Figure 2B, left panel). HT-29/siS100A10 or double knockdown of S100A10 and annexin A2 (HT-29/[siS100A10 + siANXA2]) had no obvious influence on cell proliferation (Figure 2B). Silencing of the S100A10 or annexin A2 gene had little effect on sensitivity to L-OHP in HT29 cells (Figure 2C, left panel). Double knockdown of S100A10 and annexin A2 showing a similar pattern of protein expressions as single knockdown of annexin A2 also showed little difference on sensitivity to L-OHP (Figure 2C, right panel).

### Relationships between the mRNA and protein expression levels of S100A10 and annexin A2

**Figure 3:**
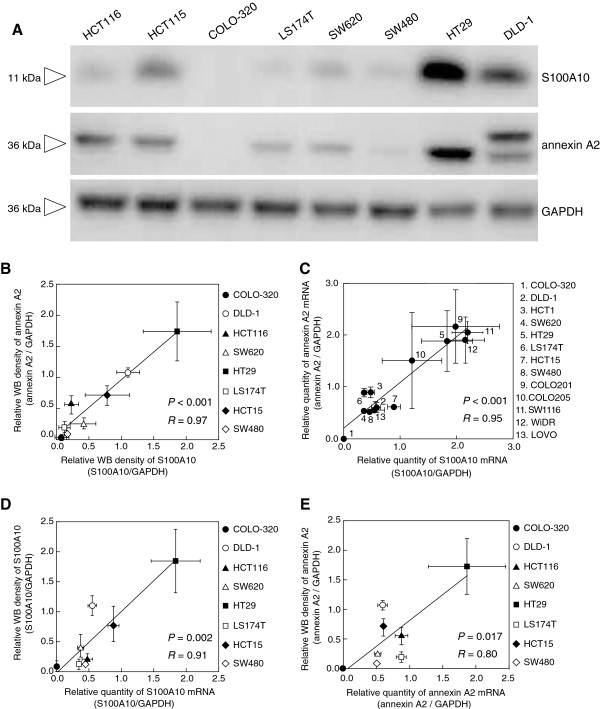
**Relationships between protein and mRNA expression levels of S100A10 and annexin A2.** The expression levels of S100A10 and annexin A2 were examined by western blot analyses **(A, B, D, E)** and real-time qRT-PCR **(C, D, E)**. **(A)** A western blot illustrating the differential expression of S100A10 and annexin A2 derived from cell lysates of 8 CRC cell lines. The results are representative of 3 separate experiments. S100A10 and annexin A2 protein expression levels in 8 CRC cell lines **(B)**, S100A10 and annexin A2 mRNA expression levels in 13 CRC cell lines **(C)**, mRNA and protein expression levels of S100A10 in 8 CRC cell lines **(D)**, and mRNA and protein expression levels of annexin A2 in 8 CRC cell lines **(E)** were investigated by Pearson’s correlation coefficient test. There was a significant and positive correlation between S100A10 and annexin A2 mRNA expression levels **(C)**, and S100A10 protein levels positively correlated with S100A10 mRNA levels **(D)**. Data were plotted as the mean ± SD (n = 3). Western blot densitometry and relative quantity of S100A10 and annexin A2 transcripts are plotted as means ± SD (n = 3).

Considering the suppression of S100A10 protein levels by silencing of the annexin A2 gene, we next investigated the associations between S100A10 and annexin A2 mRNA and protein expression. The protein expression of both S100A10 and annexin A2 in whole cell lysates from 8 CRC cell lines, quantified by western blot densitometry (Figure 3A), demonstrated a statistically strong correlation (*P* < 0.001, *R* = 0.97) between the 2 targets (Figure 3B). Real-time qRT-PCR analyses also revealed a positive correlation between mRNA expression levels of S100A10 and those of annexin A2 in 13 CRC cell lines (*P* < 0.001, *R* = 0.95, Figure 3C). Furthermore, S100A10 protein expression levels were strongly correlated with S100A10 mRNA expression levels (*P* = 0.002, *R* = 0.91, Figure 3D); the same could be said of annexin A2 (*P* = 0.017, *R* = 0.80, Figure 3E).

In DLD-1 cells, annexin A2 appeared as 2 bands around 36 kDa (Figure 3A), probably representing the 2 isoforms of annexin A2, in which isoform 2 has a substitution of 19 amino acids in Met 1 of isoform 1, resulting in a molecular weight that is 2 kDa higher than that of isoform 1. The characteristics of isoform 2 are not well known; however, we evaluated the protein expression of annexin A2 in DLD-1 cells by taking the sum of the density of both bands because all the other amino acids, including all functional sequences, other than Met 1, are identical between the 2 isoforms (UniProtKB/Swiss-Prot: http://www.uniprot.org/uniprot/P07355).

## Discussion

Predictive markers of chemotherapeutic response are urgently needed to improve the outcomes of cancer treatment. Predictive markers of the response to L-OHP have not yet been established [8,30], and clinically available protein markers of drug responses are also limited [30]. Using a proteomics approach, we recently found that intracellular S100A10 protein expression levels were significantly correlated with the sensitivity of CRC cells to L-OHP, but not to 5-FU, providing new insights into predictive markers of the response to L-OHP [10]. Therefore, in the current study, we investigated whether S100A10 was involved in mediating L-OHP sensitivity by using stably transfected cells expressing S100A10 and RNA interference.

Forced expression of S100A10 in COLO-320 CRC cells, which do not express endogenous S100A10, significantly reduced the sensitivity of these cells to L-OHP. In particular, substantial changes in cell viability were observed between 1 and 10 μM of L-OHP exposure, corresponding approximately to the blood concentration of L-OHP in clinical use [31]. A roughly 4-fold change in IC_50_ value for L-OHP observed seems not to be prominent, but should be considered. First, IC_50_ values for L-OHP in this study correspond approximately to the peak plasma concentration after intravenous infusion (Cmax) in clinical setting, and Cmax is thought to be important for efficacy, considering that antitumor activity of L-OHP is concentration and time dependent [32,33] and infusion time of L-OHP is almost fixed as 2 hours except in some cases with high risk of toxicity. Second, interpatient variability in L-OHP pharmacokinetics, evaluated as ultrafiltrable platinum, is moderate to low and 4-fold change is above interpatient variability in Cmax, indicating that this magnitude of change in sensitivity is unignorable [31,34,35].

On the other hand, forced expression of S100A10 did not alter sensitivity to 5-FU, suggesting that, as a predictive marker, S100A10 is more specific to L-OHP sensitivity than to 5-FU sensitivity. These observations strongly support our recent findings that CRC cells with higher S100A10 protein expression levels exhibit lower sensitivity to L-OHP, but not to 5-FU [10]. These results were also consistent with previous studies demonstrating an association between upregulation of S100A10 and enhancement of cell viability. S100A10 interacts with Bcl-xL/Bcl-2 associated death promoter (BAD), a death enhancer, and blunts its pro-apoptotic activity [36]. S100A10 is induced by nerve growth factor, and increased S100A10 levels promote the proliferation of PC12 cells, a pheochromocytoma cell line [37]. Moreover, limbal epithelial cell proliferation and differentiation are reported to be associated with S100A10 expression [38]. S100A10 is also a potential inducer of nuclear factor-κB (NF-κB) via activation of the Akt pathway [39], which is involved in cell growth, anti-apoptotic signaling, and carcinogenesis in tumor cells [40,41]. Interestingly, S100A10 is also thought to be a target of NF-κB [42], leading the possibility of further enhancement of cell growth by S100A10. Thus, previous data suggest that S100A10 may act as an anti-apoptotic factor.

The differences in the antitumor mechanisms and/or the opposite effects exerted on NF-κB by L-OHP or 5-FU [43-46] may be involved in mediating the distinct effect of S100A10 on cellular chemosensitivities. However, the underlying mechanisms of the different effects of S100A10 on the sensitivity to each antitumor drug have not been clarified in the current study, and further studies are required to completely elucidate these mechanisms.

The interaction between S100A10 and annexin A2 may be partly involved in the mechanisms of sensitivity to L-OHP. Most of the S100A10 is tightly associated with annexin A2 dimers, forming an (S100A10)_2_-(annexin A2)_2_ heterotetramer [23-25]. S100A10 acts as a key molecule for the promotion of angiogenesis and tumor metastasis [22], and annexin A2 is believed to act as a scaffolding protein to secure S100A10 to the cell surface, considering the well-established roles of S100A10 in cellular plasmin regulation [47-49]. Conversely, annexin A2, well known to have multiple functions and biological role in cancer cells [29,50], also requires S100A10 for its action and translocation to the cell surface [51]. In this study, we observed that reduction in both S100A10 and annexin A2 protein expression, shown in HT29/siANXA2 and HT29/(siS100A10 + siANXA2) cells. The decreased proliferation rate in HT29/siANXA2 cells, probably due in part to cell cycle arrest at the G_2_ phase [52], is unlikely to influence sensitivity to L-OHP since L-OHP is cell-cycle nonspecific [53]. These findings lead us to speculate that S100A10 may function collaboratively with annexin A2 in chemoresistance, although its precise mechanisms are still unknown because siRNA knockdown experiments failed to discriminate the difference between S100A10 and annexin A2 in terms of their contributions to chemoresistance due to simultaneous changes in the expression of S100A10 and annexin A2 proteins induced by single knockdown of annexin A2. Silencing of S100A10 and/or annexin A2 showed no apparent effect on sensitivity of the cells to L-OHP. The inconsistent effects of S100A10 forced expression and RNA interference on the sensitivity to L-OHP may partly due to the difference in baseline expression of annexin A2 between COLO-320 and HT29 cells. Native COLO-320 cells express no endogeneous annexin A2 whereas HT29 cells highly express annexin A2 [10]. Another limitation of the present study is that the efficiency of siRNA transfection into cells could not be normalized since a co-transfected marker was not used. Transfection was attempted by adding siRNA-transfection reagent complex to cells in each well of 96-well plates.

Intracellular S100A10 protein expression may also be mediated by post-translational suppression possibly due to the instability of S100A10 protein induced by annexin A2 suppression, considering that annexin A2 stabilizes intracellular S100A10 via binding, which masks the S100A10 polyubiquitination signal leading to proteasomal degradation, as previously reported [54-58]. However, there was a positive correlation between S100A10 and annexin A2 mRNA expression levels (Figure 3C), and S100A10 protein levels positively correlated with S100A10 mRNA levels (Figure 3D), suggesting that the original levels of intact S100A10 protein were not necessarily attributed to annexin A2 regulation and therefore not simply a reflection or a surrogate of annexin A2 levels. In fact, Hajjar et al. reported that the expression of S100A10 in glioma cells is not affected by stable depletion of annexin A2, probably owing to binding partners of S100A10 other than annexin A2, in her response to letters to her article [59].

Additionally, it cannot be excluded that intracellular S100A10 is a surrogate of other active molecules related to cell survival after exposure to antitumor drugs since S100A10 has been shown to interact with a variety of proteins, including plasma membrane-resident receptors and channels [11-14]. Serotonin plays important role in CRC physiology, and S100A10 interacts with the 5-HT_1B_ receptor and modulates its function [14]. S100A10 may be involved in releasing pro-inflammatory cytokines, such as interleukin-6 [39], which has been suggested to promote cell growth and apoptosis-escape in colon cancer [60,61]. Downregulation of caveolin-1, which has recently attracted attention for its potential role in chemoresistance [62,63], reduces intracellular S100A10 protein expression and localization of S100A10 to caveolae in HCT116 cells, although the mechanisms involved are unknown [64].

Thus, the molecular backgrounds of S100A10 described in previous reports are partly consistent with our hypothesis that S100A10 protein expression levels may reflect cell sensitivity to L-OHP. In addition, we demonstrated that protein expression of S100A10 was involved in mediating sensitivity to L-OHP, at least in part, by using stably transfected cells expressing S100A10. However, the mechanisms of S100A10 involvement in chemoresistance are still unknown, and the suppression of S100A10 protein expression induced by knockdown of annexin A2 makes it difficult to differentiate between the effects of annexin A2 and those of S100A10 on chemosensitivity. In the present study, we have demonstrated that protein expression of S100A10 was associated with resistance to L-OHP, but not 5-FU. However, further studies are required in order to fully elucidate the molecular mechanisms through which S100A10 acts as a predictive biomarker of the response to L-OHP.

## Conclusions

We have shown that protein expression of S100A10 was associated with resistance to L-OHP, but not 5-FU, using forced expression of S100A10 in CRC cells. Our results provide basic findings for S100A10 as a predictive marker of the response to L-OHP. Further clinical validation and functional analysis are required to confirm this hypothesis and to elucidate the underlying biological mechanisms.

## Methods

### Agents and antibodies

L-OHP and 5-FU were purchased from WAKO Chemicals (Tokyo, Japan) and Sigma-Aldrich (St. Louis, MO, USA), respectively. Purified mouse anti-annexin II Light Chain (S100A10) monoclonal antibodies (mAbs) and purified mouse anti-human annexin II (annexin A2) mAbs were obtained from BD Biosciences (Mississauga, ON, Canada). Anti-human glyceraldehyde-3-phosphate dehydrogenase (GAPDH) mAbs were obtained from Life Technologies (Carlsbad, CA, USA). All other chemicals and reagents were of the highest purity available.

### Cell culture

DLD-1, LOVO, HT29, SW480, SW1116, WiDR, and HCT116 cells were purchased from the European Collection of Cell Cultures (Salisbury, UK), and SW620 cells were purchased from the American Type Culture Collection (Manassas, VA, USA). COLO205, HCT-15, and LS174T cells were provided by the Cell Resource Center for Biomedical Research, Tohoku University (Sendai, Japan). COLO201 cells were provided by the Japanese Collection of Research Bioresource (Tokyo, Japan), and COLO-320 cells were provided by RIKEN Bio-Resource (Tsukuba, Japan). The cells were cultured in RPMI 1640 medium supplemented with 10% fetal bovine serum (FBS) and 2 mM glutamine at 37°C in humidified air containing 5% CO_2_. Cells were used when in the exponential growth phase.

### S100A10 expression vectors and generation of a stable transfectant expressing S100A10

The Flexi HaloTag clone pFN21AB8860, an S100A10 expression vector capable of producing N-terminally HaloTag-fused recombinant S100A10 protein, was obtained from Kazusa DNA Research Institute (Kisarazu, Japan). The S100A10-nonexpressing CRC cells, that is, COLO-320 cells, were plated at a density of 3 × 10^5^ cells per 35-mm dish 24 h prior to transfection. Cells were transfected with either S100A10 in the pFN21AB8860 (COLO-320/S100A10) or with the vector control (COLO-320/vector) using TransIT-LT1 transfection reagent (Mirus, WI, USA) according to the manufacturer’s instructions. Cells stably expressing S100A10 were selected with 0.8 mg/mL of geneticine (G418 disulfate, Sigma-Aldrich) and subsequent subcloning. COLO-320/S100A10 cells were maintained in medium containing 0.4 mg/mL G418 disulfate. Stable expression of S100A10 in the cells was verified using western blot analysis.

### Small-interfering RNA (siRNA) “knockdown” experiment

S100A10 siRNA (Stealth Oligo ID: 143791), annexin A2 siRNA (Stealth Oligo ID: 179173), and nontargeting control siRNA (Silencer Select Negative Control #1) were purchased from Life Technologies. Twenty-four hours prior to transfection, HT29 cells were plated at a density of 5 × 10^3^ cells/well in 96-well plates for cell proliferation assays, chemosensitivity tests, and mRNA expression analyses and were plated at 1.5 × 10^5^ cells/well in 6-well plates, scaling up proportionally to the relative surface area of culture vessels, to prepare cell lysates for western blot analysis. Cells were transfected with appropriate siRNAs for 24 h by using Lipofectamine RNAiMAX (Life Technologies) as described in the manufacturer’s standard protocol. The final siRNA concentration was 10 nM for single-knockdown experiments and 20 nM for double-knockdown experiments (concentration of each siRNA: 10 nM). After transfection, cells were processed for subsequent chemosensitivity test. siRNA-mediated knockdown of target molecules was confirmed by quantitative real-time reverse transcription (qRT)-PCR and western blot analysis at 48 h after transfection.

### Cell proliferation assay and chemosensitivity test

The viability of cells in the cell proliferation assay and chemosensitivity test in stably transfected COLO-320 cells was assessed by using the CellTiter96 AQueous One Solution Cell Proliferation Assay (MTS assay, Promega Corporation, Madison, WI, USA) according to the manufacturer’s protocol. Cell proliferation was measured 4 days after plating cells at a density of 0.5 × 10^3^ cells/well in 96-well plates. For chemosensitivity tests, cells were plated at a density of 0.5 × 10^3^ cells/well in 96-well plates 24 h prior to exposure to L-OHP (0, 0.01, 0.1, 1, 10, or 100 μM) or 5-FU (0, 0.1, 1, 10, 100, or 1000 μM). Cell viability was assessed after 48 h exposure to L-OHP or 72 h of exposure to 5-FU, and the 50% inhibitory concentration (IC_50_) was calculated from graphical plots.

Crystal violet staining (CVS) was used to determine cell numbers for assessing the cell viability of HT29 cells in siRNA-mediated knockdown experiments. Cell proliferation was measured at day 0 (just before transfection), day 3 and day 5, that is, 48 and 96 h after the end of transfection, respectively. For chemosensitivity tests, cells transfected with appropriate siRNAs for 24 h were subsequently exposed to L-OHP (0, 0.001, 0.01, 0.1, 0.3, 1, 3, 10, 30, 100, or 1000 μM) for 24 h. The medium was then changed, and cell survival was assessed by CVS after 24 h of incubation. The CVS was performed by the modified method of Sergent et al. [65]. Briefly, surviving cells were fixed by exposure to pure ethanol, stained for 30 min with 0.1% crystal violet solution in 10% ethanol, and washed with abundant tap water. After air-drying, the dye was eluted with 33% acetic acid, and the optical density of dye extracts was measured at 540 nm. The percentage of surviving cells was determined, and the IC_50_ values were calculated.

The IC_50_ values for L-OHP or 5-FU were log transformed for normal distribution, and the log_10_ IC_50_ values were used for further statistical analysis.

### Preparation of cell lysates and western blot analysis

To investigate the relationship between S100A10 and annexin A2 protein expression, 8 CRC cell lines (HCT116, HCT15, COLO-320, LS174T, SW620, SW480, HT29, and DLD-1) were used. Cells were scraped off 100-mm dishes into a lysis buffer containing 9 M urea, 2% CHAPS, 1 mM dithiothreitol (DTT), and a protease inhibitor cocktail (Sigma-Aldrich). After incubation on ice for 10 min followed by sonication on ice, the lysates were cleared by centrifugation at 15,000 × *g* for 30 min at 4°C, and protein concentrations were determined by the DC protein assay (Bio-Rad Laboratories, Hercules, CA, USA). In experiments using stably transfected COLO-320 cells or RNA interference, cell lysates were prepared by using M-PER Mammalian Protein Extraction Reagent (Thermo Fisher Scientific Inc., Rockford, IL, USA) with 1 mM DTT, 0.1 mM phenylmethylsulfonyl fluoride, and a protease inhibitor cocktail (Sigma-Aldrich) according to the manufacturer’s protocol. After centrifugation at 15,000 × *g* for 10 min at 4°C to remove cellular debris, protein concentrations were determined by the Pierce BCA protein assay (Thermo Fisher Scientific Inc.), and aliquots were quickly frozen in liquid nitrogen and stored at −80°C until analysis in all experiments.

Cell lysates were separated by SDS-polyacryamide gel electrophoresis. The separated proteins were transferred electrophoretically to polyvinylidene difluoride membranes and probed with the respective primary antibodies and alkaline phosphatase-conjugated secondary antibodies (Life Technologies) as described previously [10]. GAPDH was used as a loading control. Protein bands were visualized with an LAS 4000 mini imaging system and analyzed with Multi Gauge software ver. 3.0 (FUJIFILM, Tokyo, Japan).

### RNA extraction and real-time qRT-PCR

For quantification of mRNA expression, cells were plated at a density of 5 × 10^3^ cells/well in 96-well plates in all experiments. Extraction of total RNA from cultured cells and synthesis of cDNA from total RNA were performed using the TaqMan Gene Expression Cells-to-CT Kit (Life Technologies) according to the manufacturer’s instructions. The real-time qRT-PCR measurement of individual cDNAs was performed using TaqMan Gene Expression Assays for S100A10 (Assay ID: Hs00237010_m1), annexin A2 (Assay ID: Hs00237010_m1), and GAPDH (Assay ID: Hs99999905_m1) on an ABI 7900 Real-Time PCR System (Life Technologies). The reactions were run in 384-well plates, using the following program: 50°C for 2 min followed by 95°C for 10 min, followed by 40 cycles of 95°C for 15 s, 60°C for 1 min. The cycling parameters were manufacturer’s specifications. The relative standard curve method, preparing serial dilution of total RNA (1×, 10×, 20×, 40×, 80×, 160×, 320×, 800×, 1600×) prepared from the pool of total RNA obtained by combining aliquots of samples for all assay, was used to quantify the results obtained by real-time qRT-PCR. Relative fold-changes were normalized to the expression of GAPDH.

### Statistical analysis

Statistical analyses were performed using SPSS software 19.0 J for Windows (SPSS, Chicago, IL, USA). Comparison between groups was performed by one-way analysis of variance (ANOVA) followed by post-hoc multiple pairwise comparison using Tukey’s test to determine statistical differences. To evaluate relationships between 2 variables, Pearson’s correlation coefficient test was used. *P* values of less than 0.05 were considered statistically significant.

### Ethical approval

Our *in vitro* study described in this manuscript used the cell lines commercially available. This type of study does not apply to human subject research by standards of Guidance from US-Office for Human Research Protection (OHRP). OHRP states that “OHRP does not consider the act of solely providing coded private information or specimens (for example, by a tissue repository) to constitute involvement in the conduct of the research”.

Furthermore, NIH Office of Extramural Research in US Department of Health & Human Service (HHS) also answers to investigators in their FAQs that “Research that proposes the use of human cell lines available from American Type Culture Collection or a similar repository is not considered human subjects research because the cells are publicly available and all of the information known about the cell lines is also publicly available”. Therefore, our study does not apply to an ethics committee.

## Abbreviations

L-OHP: Oxaliplatin; CRC: Colorectal cancer; IC50: 50% inhibitory concentration; ANXA2: Annexin A2.

## Competing interests

Yusuke Tanigawara, Sayo Suzuki and Yakult Honsha Co., Ltd. hold patents on S100A10 titled as “METHOD FOR DETERMINATION OF SENSITIVITY TO ANTI-CANCER AGENT “(Patent No. 2010/05157 and 586972). This does not alter the authors’ adherence to all Proteome Science policies on sharing data and materials.

## Authors’ contributions

YT was responsible for planning and designing the study and data interpretation. SS performed the experiments and the data analysis and wrote the manuscript. Both authors read and approved the final manuscript.
